# MEMS-Based Integrated Triaxial Electrochemical Seismometer

**DOI:** 10.3390/mi12101156

**Published:** 2021-09-26

**Authors:** Wenjie Qi, Bowen Liu, Tian Liang, Jian Chen, Deyong Chen, Junbo Wang

**Affiliations:** 1 State Key Laboratory of Transducer Technology, Aerospace Information Research Institute, Chinese Academy of Sciences, Beijing 100190, China; qiwenjie16@mails.ucas.ac.cn (W.Q.); liubowen17@mails.ucas.ac.cn (B.L.); liangtian18@mails.ucas.ac.cn (T.L.); chenjian@mail.ie.ac.cn (J.C.); 2 School of Electronic, Electrical and Communication Engineering, University of Chinese Academy of Sciences, Beijing 100049, China

**Keywords:** electrochemical seismometer, integrated triaxial, micro-electro-mechanical system

## Abstract

This paper presents a micro-electromechanical systems (MEMS)-based integrated triaxial electrochemical seismometer, which can detect three-dimensional vibration. By integrating three axes, the integrated triaxial electrochemical seismometer is characterized by small volume and high symmetry. The numerical simulation results inferred that the integrated triaxial electrochemical seismometer had excellent independence among three axes. Based on the experimental results, the integrated triaxial electrochemical seismometer had the advantage of small axial crosstalk and could detect vibration in arbitrary directions. Furthermore, compared with the uniaxial electrochemical seismometer, the integrated triaxial electrochemical seismometer had similar sensitivity curves ranging from 0.01 to 100 Hz. In terms of random ground motion response, high consistencies between the developed integrated triaxial electrochemical seismometer and the uniaxial electrochemical seismometer could be easily observed, which indicated that the developed integrated triaxial electrochemical seismometer produced comparable noise levels to those of the uniaxial electrochemical seismometer. These results validated the performance of the integrated triaxial electrochemical seismometer, which has a good prospect in the field of deep geophysical exploration and submarine seismic monitoring.

## 1. Introduction

A seismometer is a kind of sensor that can transform ground vibrations caused by seismic waves into electrical signals, and is the key equipment in the field of seismology [1]. According to different working principles, seismometers are mainly divided into magnetoelectric coil seismometers, piezoelectric seismometers, capacitive seismometers, optical fiber seismometers, micro-electromechanical systems (MEMS) seismometers, and electrochemical seismometers [2,3,4,5,6,7,8,9,10,11,12]. Compared with other types, electrochemical seismometers have the advantages of wide frequency band, high sensitivity, low power consumption, large installation angle, and strong shock resistance (no vulnerable parts), which are widely used in deep geophysical explorations and ocean-bottom seismic monitoring [11,12].

The sensitive electrodes of traditional commercial electrochemical seismometers were manufactured by platinum net-waving and ceramic sinter technologies, which suffered from fatal flaws of low yield, high cost, and poor consistency [12,13,14]. To solve these problems, MEMS technology was introduced to manufacture the sensitive electrodes of electrochemical seismometers in recent years [15,16,17,18,19,20,21,22,23].

In practical applications, seismic information in three axial directions is preferred. More specifically, Chen et al. reported a monolithic electrochemical seismic microsensor based on four sensitive electrodes fixed in four vertical flow channels to realize the measurements of three-dimensional vibrations [24]. However, limited by the monolithic design, the performance of this seismometer suffered from low sensitivity and high crosstalk.

To address these issues, this paper presented a MEMS based integrated triaxial electrochemical seismometer. Leveraging current differences of three pairs of sensitive electrodes fixed in three orthogonal flow channels, the detection of three-dimensional vibration was realized. Compared with previously reported monolithic electrochemical seismic microsensors, the placement of three orthogonal flow channels in this study can greatly reduce crosstalk among three axes. In comparison to simply placing three electrochemical seismometers perpendicular to each other [12], in this study, three electrochemical seismometers were integrated into one plexiglass shell, which effectively eliminated auxiliary structures and reduced device volumes.

## 2. Structure and Working Principle

**Figure 1:**
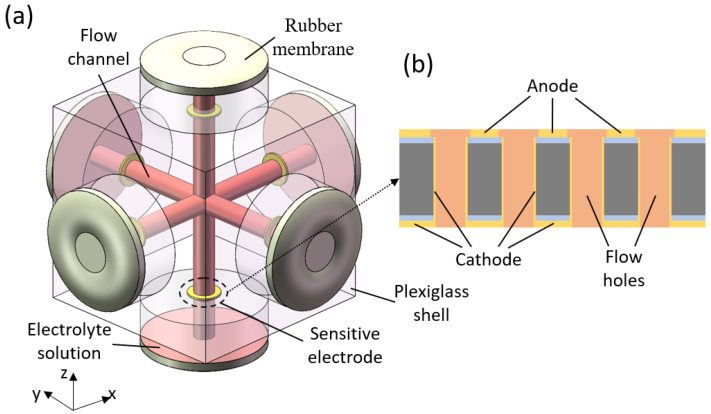
(**a**) Schematic diagram of the micro-electromechanical systems (MEMS) based integrated triaxial electrochemical seismometer developed in this study, consisting of (1) six rubber membranes, (2) six sensitive electrodes, (3) three orthogonal flow channels, (4) an electrolyte solution, and (5) a plexiglass shell; (**b**) enlarged schematic diagram of the sensitive electrode with (6) a cathode, (7) an anode, and (8) a large number of flow holes integrated on one chip.

As shown in Figure 1a, the MEMS based integrated triaxial electrochemical seismometer consists of six rubber membranes, six sensitive electrodes, three orthogonal flow channels, an electrolyte solution made of iodine and potassium iodide, and a plexiglass shell. As the key sensitive unit, the sensitive micro electrodes were composed of a cathode, an anode and a large number of flow holes, as shown in Figure 1b. Six units of sensitive electrodes were immobilized in six branches of three orthogonal flow channels, which were filled with the electrolyte solution and sealed by six rubber membranes.

**Figure 2:**
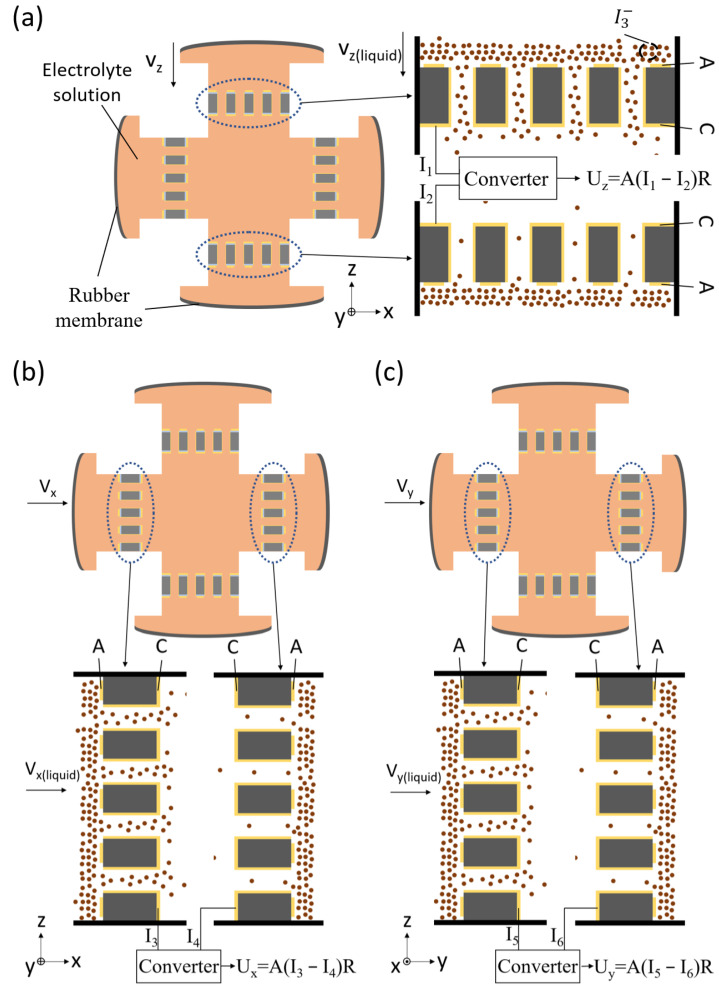
Working principle of the MEMS based integrated triaxial electrochemical seismometer, including (**a**) the working principle of the z-axis, (**b**) the working principle of the x-axis and (**c**) the working principle of the y-axis. In response to a vibration with an arbitraty direction, it was decoupled into x, y, and z directions, accordingly. Then, corresponding vibrations along individual axis lead to movements of electrolyte solution and ion movements around sensitve electrodes, which were translated into current outputs.

Figure 2 shows the working principle of the MEMS based integrated triaxial electrochemical seismometer. In response to a vibration with an arbitrary direction, it was decoupled into x, y, and z directions, accordingly due to a triaxial orthogonal flow channel.

Figure 2a shows the working principle of the z-axis, where the brown dots represent triiodide ions. In response to the vibration along the z direction, the plexiglass shell and sensitive electrode vibrate with the ground, while the electrolyte solution flows relative to the sensitive electrode due to inertia. Thus, the external vibration is converted into the flow of the electrolyte solution relative to the sensitive electrode.

In the mixed solution of iodine and potassium iodide, there is a complexation process:

(1) $$I_{2}+I^{-}=I_{3}^{-}$$

Since the concentration of iodine is much lower than that of potassium iodide, iodine is completely converted to a triiodide ion. When a DC (direct current) bias is applied between the anode and cathode, electrochemical reactions occur on the anode and cathode, respectively [25]:

(2) $$\mathrm{Anode}:\;3I^{-}-2e^{-}\to I_{3}^{-}$$

(3) $$\mathrm{Cathode}:\;I_{3}^{\to }+2e^{-}\to 3I^{-}$$

A triiodide ion is produced near the anode and consumed near the cathode, and diffuses from high concentration to low concentration. Eventually, the whole process reaches dynamic equilibrium where the concentration of triiodide ion is extremely low near the cathode. When there is no vibration, since two sensitive electrodes of z axis are completely symmetrical, the concentration distribution of triiodide ion near two sensitive electrodes is completely symmetrical, so the output current of two cathodes is equal. After current/voltage conversion and differential amplification, the output voltage of the z-axis is zero. Similarly, the output voltage of x and y axes is zero.

When the external acceleration is applied to the electrochemical seismometer along the z-axis, the seismometer responds to the external vibration, causing the electrolyte solution to flow in the flow channel of the z-axis. When the solution flows down, due to the large amount of triiodide ions carried by the solution flow, the concentration of triiodide ion around the upper cathode increases, which increases the electrochemical reaction rate of the upper cathode and leads to the increase of the cathode current $I_{1}$. As a result of the solution flow, the concentration of triiodide ion around the lower cathode decreases, which reduces the electrochemical reaction rate of the lower cathode and leads to the decrease of the cathode current $I_{2}$. After current/voltage conversion and differential amplification, the output voltage of the z-axis is:

(4) $$U_{z}=A\left(I_{1}-I_{2}\right)R$$

where *R* represents the converter resistance, *A* represents the differential amplification factor, and $I_{1}$ and $I_{2}$ represent the output currents of the upper and lower cathodes, respectively.

Figure 2b,c show the working principle of the x-axis and y-axis, which is the same as the z-axis.

## 3. Numerical Simulation

**Figure 3:**
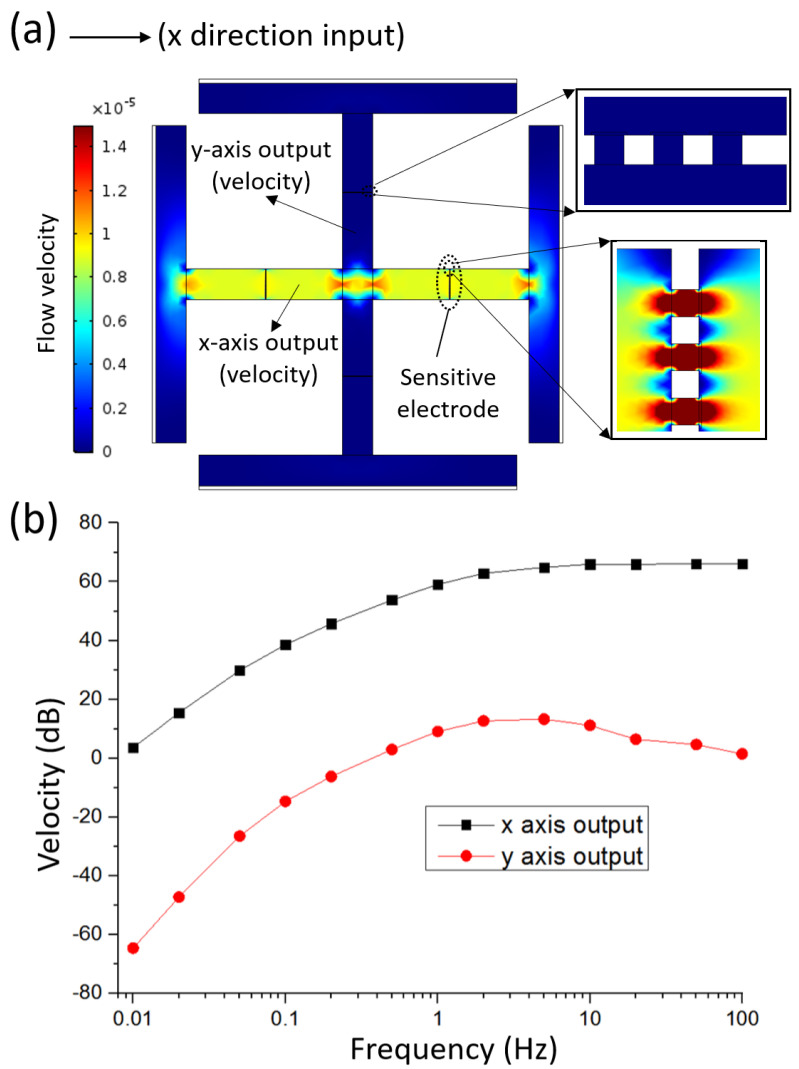
(**a**) Two-dimensional numerical simulation of the MEMS based integrated triaxial electrochemical seismometer in response to the x-direction vibration. (**b**) The simulation results of the MEMS based integrated triaxial electrochemical seismometer where multi-frequency velocities in the x-direction were inputting parameters and velocities on the x and y axes were outputting parameters.

In order to better study the characteristics of the MEMS based integrated triaxial electrochemical seismometer, this paper used the finite element simulation (COMSOL Multiphysics 5.3, Stockholm, Sweden) to verify the independence of three axes and check the decoupling ability of the seismometer to the vibration in an arbitrary direction. Figure 3a shows the two-dimensional simplified simulation model of the vibration picking module of the proposed seismometer, and the enlarged view of the sensitive electrode is on the right. In this simulation, the “fluid-structure coupled physical field” was used to convert the input velocity to the flow velocity of the solution in the flow channel.

Figure 3a,b shows the numerical simulation of the integrated triaxial electrochemical seismometer where multi-frequency velocities in the x direction were inputting parameters and velocities at x and y axes were outputting parameters. Due to the second-order high-pass filtering characteristics of the vibration picking module, the increase of the inputting frequency was observed to increase the output velocities on both the x and y axes. In addition, it can be clearly observed that the output of the y axis was much smaller than that of the x axis, which indicates the independence of the detection units of x and y axes.

In conclusion, the simulation results show that the x and y axes have good independence, and it can be inferred that the three axes of the integrated triaxial electrochemical seismometer also have similar properties.

## 4. Fabrication

**Figure 4:**
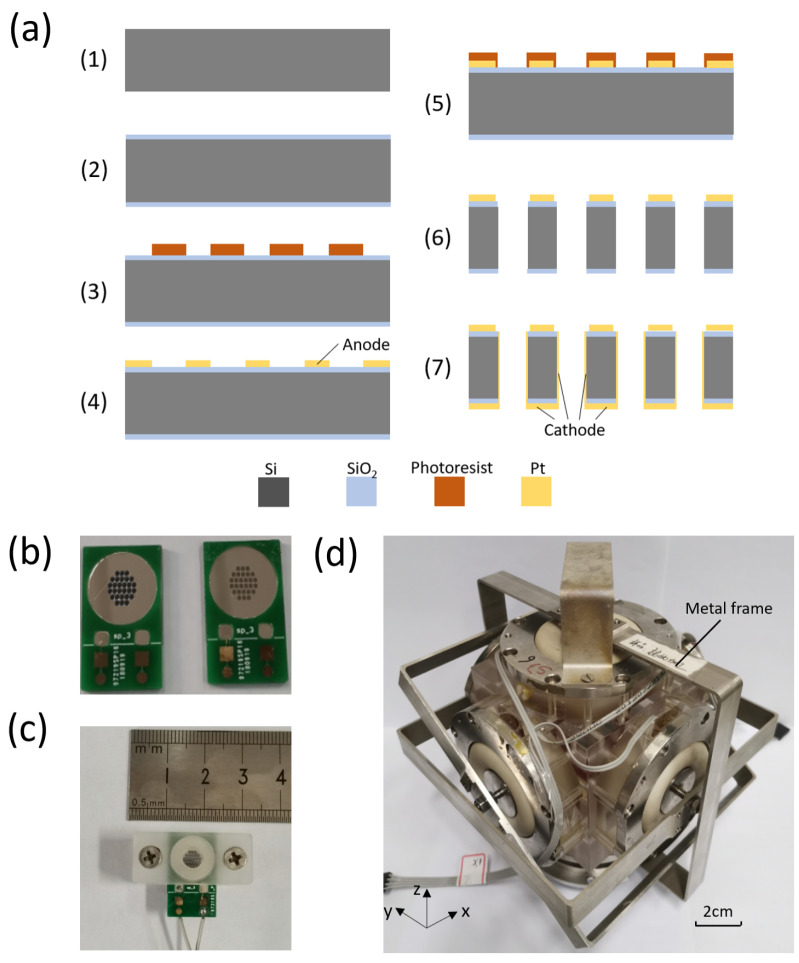
Manufacturing process of sensitive electrode (**a**) and fabrication process of the integrated MEMS based triaxial electrochemical seismometer (**b**–**d**) with a prototype device shown in (**d**).

Figure 4a shows the fabrication process of the sensitive electrode:

(1) The silicon wafer (200 microns in thickness) was put into the concentrated sulfuric acid mixed with hydrogen peroxide, heated to 120 °C, and then kept for 30 min. After cooling, the silicon wafer was put into deionized water and heated to boil.
(2) The silicon wafer was thermal oxidized to produce a 600 nm oxide layer on both sides.
(3) Photoresist AZ5214E was spin-coated on the front of the silicon wafer, and then exposed and developed (developer: 0.6% NaOH) to form a patterned photoresist. Then, the wafer was cleaned with oxygen plasma (30 s).
(4) Titanium (Ti) (40 nm in thickness) and platinum (Pt) (200 nm in thickness) were sputtered on the patterned photoresist in sequence. Then, the patterned anode was formed by a lift-off technique, and the residual photoresist was then removed with acetone.
(5) Photoresist AZ4620 was spin-coated on the topside of the silicon wafer, and then exposed and developed (developer: 1% NaOH) to form a patterned photoresist. Then, the wafer was cleaned with oxygen plasma (30 s).
(6) Using the patterned photoresist as a mask, reactive ion etching technology (RIE) and deep reactive ion etching technology (DRIE) were used to etch the silicon wafer to form a large number of flow holes (80 microns in diameter). The residual photoresist was then removed with acetone.
(7) Titanium (Ti) (40 nm in thickness) and platinum (Pt) (200 nm in thickness) were successively sputtered on the backside of the silicon wafer to fabricate the cathode, in which the surface of the wafer and the side walls of the flow holes were sputtered with electrode.

Figure 4b–d shows the assembly process of the MEMS based integrated triaxial electrochemical seismometer with a prototype device included. Figure 4b shows two sides of the sensitive electrode (attached to PCB), with the anode on the left and the cathode on the right. Figure 4c shows the assembled sensitive electrode, and Figure 4d shows the assembled prototype device. The triaxial flow channel and six surfaces of the triaxial device were sealed by mechanical compaction, while six sensitive electrodes were fixed in the middle of six branches of triaxial orthogonal flow channels. In addition, to enable the device to work properly, a spring was placed at the bottom to support the gravity of the electrolyte solution, and three supporting frames were placed on three axes to ensure that three pairs of rubber membranes were in good working condition.

## 5. Devices’ Characterizations

In order to verify the performance of the MEMS-based integrated triaxial electrochemical seismometer, sensitivity curves of three axes were calibrated and compared with that of the uniaxial electrochemical seismometer. Then, the output and crosstalk of the integrated triaxial electrochemical seismometer in response to vibration along three axes were measured, in comparison with that of the conventional triaxial electrochemical seismometer simply placing three electrochemical seismometers perpendicular to each other. In addition, the decoupling ability of the integrated triaxial electrochemical seismometer to vibration in an arbitrary direction was tested. Finally, the test result in response to random ground motion of the integrated triaxial electrochemical seismometer were compared with that of the uniaxial electrochemical seismometer.

### 5.1. Sensitivity Characterization

Sensitivity curves of the electrochemical seismometer were calibrated on an ultra-low frequency vibration table (National Institute of Metrology of China). Vibrations of 20 frequencies ranging from 0.01 Hz to 50 Hz were applied to the electrochemical seismometer, and then the output voltage of the electrochemical seismometer was measured to calculate the sensitivity at different frequencies. In this paper, the anode was applied with 0.3 V while the cathode was connected to the virtual ground. In addition, the electrolyte solution was a mixture of 2 mol/L potassium iodide and 0.02 mol/L iodine. Moreover, the resistance of the current-voltage converter was 1 kΩ, and the differential amplification factor was 3.4.

**Figure 5:**
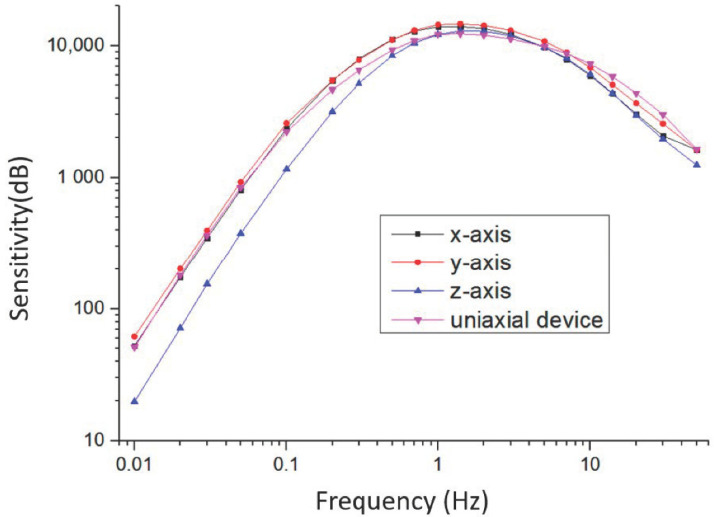
Measurement results of the sensitivity curves of x-, y-, and z-axes of the integrated triaxial device in comparison with the sensitivity curves of the uniaxial device.

Figure 5 shows the sensitivity curves of x-, y-, and z-axes of the integrated triaxial device in comparison with the sensitivity curves of the uniaxial device (placed along the x direction). The peak sensitivities of x-, y-, and z-axes were 13,961 V/(m/s), 14,657 V/(m/s) and 12,925 V/(m/s) at 1.4 Hz, respectively, and the peak sensitivity of the uniaxial device was 12,355 V/(m/s) at 1.4 Hz. It can be clearly observed that the sensitivity curves of x- and y-axes were close to that of the uniaxial device. As for the z-axis, due to the influence of spring, the elastic coefficient of z-axis increases, which moves the center frequency of the vibration picking module to high frequency, thus reducing the sensitivity at low-frequency. The test results show that three axes of the integrated triaxial device can work as well as the uniaxial device. In addition, the performance comparison between the electrochemical seismometer similar to the uniaxial device and the commercial device CME6011 has been discussed in previous articles (see in reference [23,24]).

### 5.2. Axial Crosstalk Coefficient

**Figure 6:**
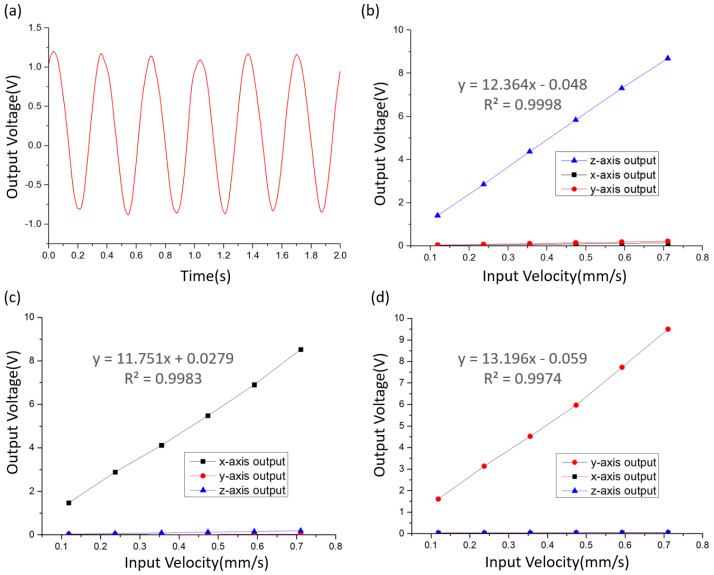
(**a**) The output voltage of the z-axis of the developed integrated triaxial electrochemical seismometer under the input velocity of 0.08 mm/s at 3 Hz along the z direction; (**b**–**d**) the output voltage of x-, y-, and z-axes of the developed integrated triaxial electrochemical seismometer by applying independent vibration of different amplitudes along x, y, and z directions.

Axial crosstalk refers to the phenomenon that vibration input along one direction produces output on another axis. In the experiment of measuring the axial crosstalk coefficient of the MEMS-based integrated triaxial electrochemical seismometer, the output voltage of three axes was measured by applying independent vibration of different amplitudes in the x, y, and z directions, and then the corresponding axial crosstalk coefficient was calculated (see Figure 6). In order to make the results more representative, the frequency of the input velocity was selected as 3 Hz, near the peak sensitivity.

Figure 6b–d show the output voltage of x-, y-, and z-axes of the developed integrated triaxial electrochemical seismometer by applying independent vibration of different amplitudes along x, y, and z directions. For example, under the input velocity of 0.08 mm/s at 3 Hz along the z direction, the time domain diagram of the z-axis of the developed integrated triaxial electrochemical seismometer is shown in Figure 6a.

Figure 6b shows the output voltage of three axes when the vibration of different amplitudes was applied along the z direction. It is obvious that the output voltage of x- and y-axes was much lower than that of the z-axis. According to the sensitivity test results, the sensitivities of x-, y-, and z-axes (Sx, Sy, Sz) at 3 Hz were 11,751 V/(m/s), 13,196 V/(m/s), and 12,364 V/(m/s), respectively. After calculation, the crosstalk coefficients of the x-axis ((Ux/Sx)/Vz) and y-axis ((Uy/Sy)/Vz) in response to the vibration along the z direction were 1.59% and 2.32%, respectively, where Ux is the output voltage of the x-axis, Uy is the output voltage of the y-axis, and Vz is the input velocity of the z direction.

Figure 6c shows the output voltage of three axes when the vibration of different amplitudes was applied along the x direction. Obviously, the output voltage of y- and z-axes was much lower than that of the x-axis. After calculation, the crosstalk coefficients of the y-axis ((Uy/Sy)/Vx) and the z-axis ((Uz/Sz)/Vx) in response to the vibration along the x direction were 0.46% and 2.11%, respectively, where Uz is the output voltage of the z-axis and Vx is the input velocity of the x direction.

Figure 6d shows the output voltage of three axes when the vibration of different amplitudes was applied along the y direction. It can be clearly observed that the output voltage of the y- and z-axes was much lower than that of the x-axis. After calculation, the crosstalk coefficients of the x-axis ((Ux/Sx)/Vy) and y-axis ((Uz/Sz)/Vy) in response to the vibration along the y direction vibration were 0.55% and 0.64%, respectively, where Vy is the input velocity of the y direction.

Based on these data, it can be seen that the crosstalk coefficient of x- and y-axes in response to the vibration along the z direction was large, which was mainly caused by the fact that the z-axis was not completely perpendicular to the vibration platform. If there is a small angle between the z-axis and the z direction on the xz plane, setting as 1°, the vibration along the z direction will cause a vibration along the x-axis with an amplitude of 1.75%, resulting in a 1.75% axial crosstalk coefficient of the x-axis in response to the vibration along the z direction. Therefore, a small angle between the z-axis and z direction can cause a large axial crosstalk coefficient of x and y-axes in response to the vibration along the z direction.

In addition, the axial crosstalk coefficient of the z-axis in response to the vibration along the x direction was large, while the interaxial crosstalk coefficient of the z-axis in response to the vibration along the y direction was much smaller. After many tests, the reason was found: the z-axis of the integrated triaxial device had a support frame. When the vibration was applied along the direction of the support frame, there was a large crosstalk output on the z-axis; when the vibration was applied along the direction perpendicular to the support frame, the crosstalk output of the z-axis was much smaller, which explains the above phenomenon. More specifically, as there was a spring at the bottom of the z-axis, and the upper and lower regions of the z-axis were asymmetrical. Therefore, the common mode crosstalk caused by x-direction vibration cannot be completely eliminated by differences, resulting in the large crosstalk output of the z-axis in response to the vibration along the x direction. To solve this problem, a stretched spring can be placed on the top of the z-axis to support the gravity of the electrolyte solution together with the bottom spring, so that the z-axis is completely symmetric, thus greatly reducing the axial crosstalk.

As a comparison, the output voltage of the uniaxial device in response to the vibration along the x and y directions was also measured. Among them, the x-axis, y-axis, and z-axis devices were the same uniaxial device placed along the x, y, and z directions, respectively. In addition, the frequency of the input velocity was 3 Hz.

**Figure 7:**
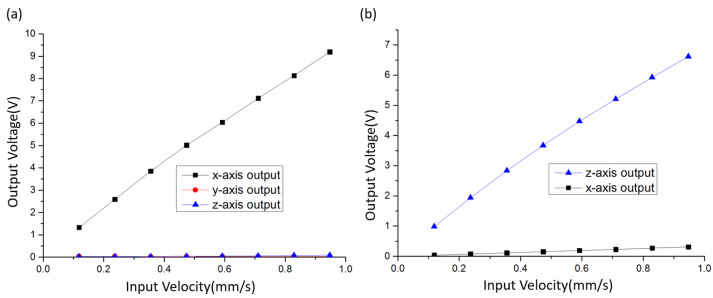
The output voltage of x-axis, y-axis, and z-axis devices when the vibration of different amplitude was applied along x, y (**a**), and z directions (**b**), respectively.

Figure 7a shows the output voltages of x-axis, y-axis, and z-axis devices in response to the vibration along the x direction. It can be clearly observed that the output voltage of y-axis and z-axis devices was much lower than that of the x-axis device. According to the sensitivity test results, the sensitivity of the x-axis device (Sx) at 3 Hz was 12,212.4 V/(m/s). After calculation, the crosstalk coefficients of the y-axis device ((Uy/Sy)/Vx) and z-axis device ((Uz/Sz)/Vx) in response to the vibration along the x direction were 0.72% and 0.77%, respectively.

Figure 7b shows the output voltage of x-axis and z-axis devices in response to the vibration along the z direction. Obviously, the output voltage of the x-axis device was much lower than that of the z-axis device. The test results show that the sensitivity of the z-axis device (Sz) at 3 Hz was 8321.5 V/m/s. After calculation, the crosstalk coefficient of the x-axis ((Ux/Sx)/Vz) in response to the vibration along the z direction was 2.66%. Since the x axis and y axis are only defined differently, there is no need to test the other four groups of data.

For the conventional triaxial electrochemical seismometer, simply placing three electrochemical seismometers perpendicular to each other, three electrochemical seismometers are orthogonal by means of auxiliary structure, and the assembly structure is relatively complex. Machining errors and assembly errors of many components will lead to the deviation of orthogonality between three axes, while a small angle deviation can lead to a large axial cross-talk coefficient. Therefore, the axial cross-talk coefficient of the conventional triaxial electrochemical seismometer is larger than that of the uniaxial device.

In conclusion, three orthogonal flow channels machined in the integrated triaxial device accurately guarantee the orthogonality between three axes, thus reducing the axial crosstalk. Due to the existence of spring, the crosstalk of the z-axis in response to the vibration along x and y directions was increased.

### 5.3. Decoupling Test

In order to test the decoupling capability of the integrated triaxial electrochemical seismometer for vibration in arbitrary direction, this paper tested the x- and y- axes of the integrated triaxial device in response to the input vibration of different amplitudes at 45 degrees of the x direction (xy plane).

**Figure 8:**
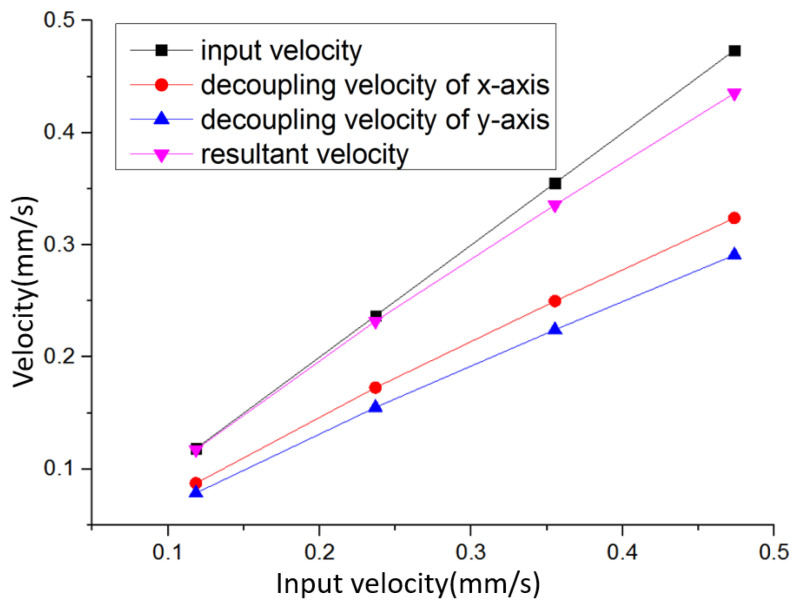
Measurement results of the developed integrated triaxial electrochemical seismometer in response to the input vibration with different amplitudes at 45 degrees of the x direction (xy plane).

Figure 8 shows the test results, in which the decoupling velocity of x- and y- axes was converted from the output voltage. When the input velocity was small, such as 0.12 mm/s, the resultant velocity was very close to the input velocity. When the input velocity was large, there was a relatively large deviation between the resultant velocity and the input velocity, which was caused by the nonlinearity of the device when approaching the maximum detection range.

The test results show that the integrated triaxial device can decouple the input vibration to x- and y- axes, and realize the accurate detection of the input vibration. This proves that the integrated triaxial electrochemical seismometer can accurately detect the vibration in arbitrary directions.

### 5.4. Response to Random Ground Motions

To further demonstrate the performance, the integrated triaxial electrochemical seismometer developed in this study and the uniaxial electrochemical seismometer were positioned side by side on the laboratory to monitor random vibrations. In addition, the x-axis of the integrated triaxial device and the uniaxial device were placed along the same direction (east–west direction). To minimize the impact of external noise, the devices were tested late at night.

**Figure 9:**
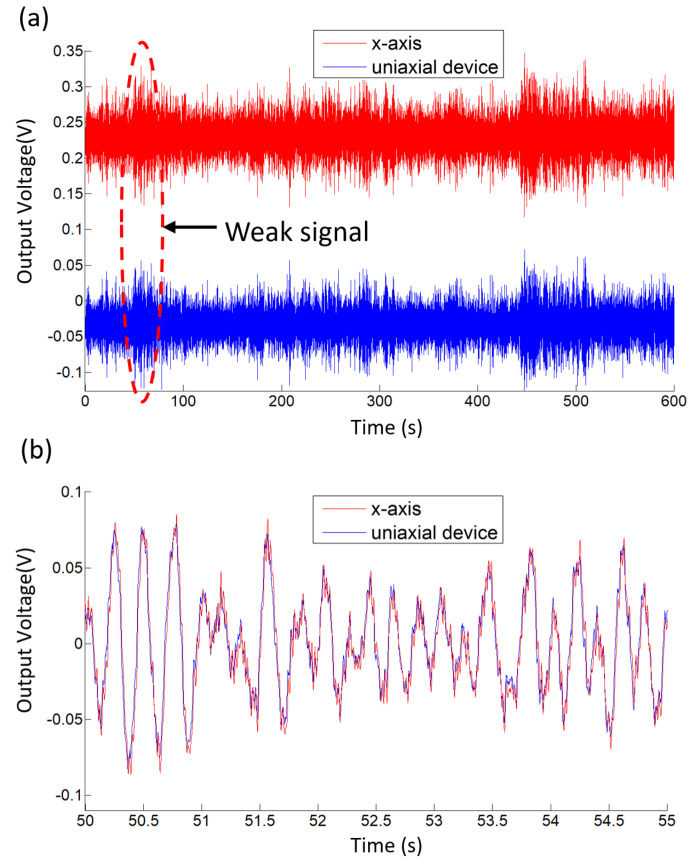
Test results of the integrated triaxial electrochemical seismometer developed in this study in comparison with the uniaxial electrochemical seismometer without observable ground motions, including (**a**) the time-domain response and (**b**) enlarged view of the normalized time-domain response from 50 to 55 s.

Figure 9a shows the time-domain response of the x-axis of the integrated triaxial electrochemical seismometer and the uniaxial electrochemical seismometer, and the corresponding correlation coefficient was quantified as 0.9639. Figure 9b shows the enlarged view of the time-domain response from 50 to 55 s, where a high consistency between the x-axis and the uniaxial electrochemical seismometer can be easily observed. The results demonstrate that the integrated triaxial electrochemical seismometer can work as well as the uniaxial electrochemical seismometer.

**Figure 10:**
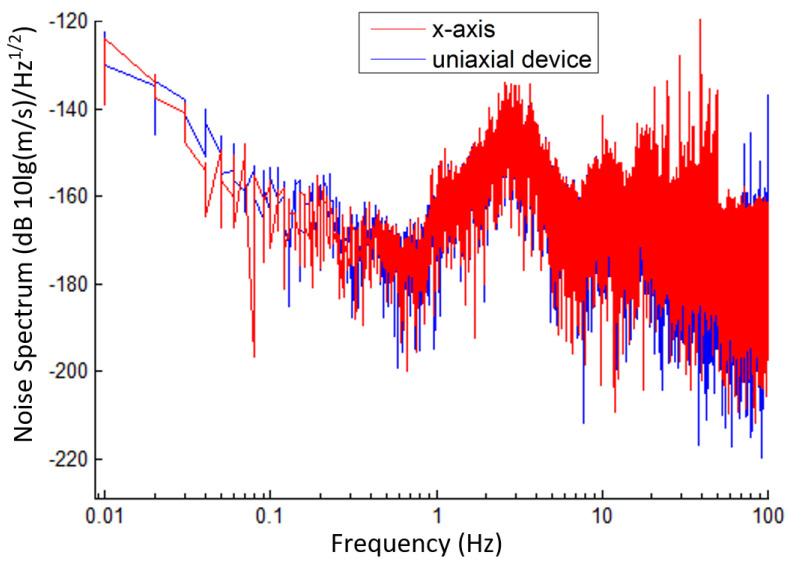
The noise power spectrum of the developed integrated triaxial electrochemical seismometer in comparison with the reference device.

Figure 10 shows the noise power spectrum of the x-axis of the integrated triaxial electrochemical seismometer in comparison with the uniaxial electrochemical seismometer. As shown in the figure, the developed electrochemical seismometer and the uniaxial electrochemical seismometer show extremely high consistency in the full frequency range of 0.01–100 Hz. Considering the high consistency of the time-domain responses, it can be concluded that the noise level of the integrated triaxial electrochemical seismometer and the uniaxial electrochemical seismometer was much lower than the noise levels of ground vibrations, which validated the low noise performance of the integrated triaxial electrochemical seismometer.

Of course, the integrated triaxial device also has some disadvantages. The design of connected triaxial flow channel brings troubles for injection and packaging, and also increases the crosstalk between three axes (in comparison with the unconnected triaxial flow channel). Furthermore, because three axes of the integrated triaxial device are integrated, the failure rate of the integrated triaxial device is higher than that of the uniaxial device.

## 6. Conclusions

This paper introduces an integrated triaxial electrochemical seismometer, which can detect three-dimension vibration directly. The experimental results show that the integrated triaxial electrochemical seismometer had excellent independence among three axes, and can accurately detect the vibration in arbitrary direction. Compared with the uniaxial electrochemical seismometer, the developed integrated triaxial electrochemical seismometer had similar sensitivity curves, and produced a comparable noise level. In conclusion, these results validated the feasibility of the integrated triaxial electrochemical seismometer developed in this study.
